# Extended experience in parieto-occipital expansion surgery by meander technique—clinical and radiological evaluation

**DOI:** 10.1007/s00381-021-05355-w

**Published:** 2021-09-16

**Authors:** Valentina Pennacchietti, Matthias Schulz, Anna Tietze, Karin Schwarz, Ulrich-Wilhelm Thomale

**Affiliations:** 1 Pediatric Neurosurgery, Charité-Universitätsmedizin Berlin, Freie Universität Berlin, Humboldt-Universität Zu Berlin, Berlin Institute of Health, Augustenburger Platz 1, 13353 Berlin, Germany; 2 Institute of Neuroradiology, Charité-Universitätsmedizin Berlin, Freie Universität Berlin, Humboldt-Universität Zu Berlin, Berlin Institute of Health, Berlin, Germany

**Keywords:** Posterior cranial expansion, Posterior plagiocephaly, Brachycephaly, Pansynostosis, Parieto-occipital remodeling

## Abstract

**Introduction:**

Brachycephaly and anterior and posterior plagiocephaly appear as an isolated entity or manifest in syndromic conditions. In severe cases, possible treatment options currently comprise either cranioplasty or osteogenetic distraction. The aim of this paper is to retrospectively review the perioperative course of a series of children treated by posterior meander expansion technique at our institution with focus on the course of postoperative intracranial volume and eventual tonsillar descent evolution.

**Methods:**

Forty-two children received a posterior cranial vault remodeling by means of a posterior meander technique during a 7-year period. Hospital records were reviewed, and pre- and postoperative MRIs were analyzed for intracranial volume, cephalic and asymmetry index, and tonsillar position over time.

**Results:**

Median age at surgery was 11.5 months (range 17 days–10 years). Nineteen children had a symmetrical cranial deformity, twenty-three an asymmetrical synostosis. Half of the cohort showed a syndromic condition. Transfusions were administered in the majority (92.2%) of the cases. A significant postoperative increase of intracranial volume was present from 1188.9 ± 370.4 cm^3^ to 1324.8 ± 352.9 cm^3^ (p < 0.001). The asymmetry index showed a significant improvement postoperatively: 0.86 ± 0.06 versus 0.91 ± 0.05 (p < 0.001), while the cephalic index showed a non-statistical change (0.91 ± 0.11 versus 0.88 ± 0.08). Tonsillar herniation, bilateral or homolateral, showed no significant changes at early control, while a nonsignificant amelioration of tonsillar descent was seen among children older than 12 months at late imaging follow-up.

**Conclusion:**

Among the osteoplastic techniques, the posterior meander technique offers several advantages, such as early mobilization of the child, less bony defects, absence of implants, and a small complication rate. However, further comparative studies among different surgical techniques are needed.

## Introduction

In cases of intracranial space restricting brachycephaly, pachycephaly, and synostosis-associated posterior plagiocephaly, posterior cranial vault expansion surgery aims not only to improve cranial deformity, but also to resolve raised intracranial pressure by increasing intracranial volume, as well as improving venous outflow and CSF circulation. Several techniques have been described such as cranial vault distraction osteogenesis [1, 2], single-stage total calvarial remodeling [3], posterior flaps [4, 5], spring-assisted expansion [6], and posterior meander technique [7, 8]. Recently, early endoscopic assisted strip craniectomies to reach a posterior calvarial expansion have been described [9, 10]. Reviewing different techniques led to a postulation that the choice of surgical measure may be best tailored to the patients’ general status and age [11]. It has been demonstrated that posterior cranial vault remodeling markedly increases intracranial volume in comparison to frontoorbital advancement [12, 13]. Thus, in particular in syndromic patients, it was suggested as a first possible intervention [13, 14].

At present, outcome evaluation for parieto-occipital expansion focuses in particular on the results of distraction techniques, while authors rely either on preoperative and postoperative CT scan analysis [15–21], while others reported cephalic index [22] and other craniomorphometric parameters [23]. In our earlier experience, we reported MRI-based calculations of intracranial volume changes together with measurements of several morphometric indices including cranial index [8]. Another important factor might be the radiological development of tonsillar descent, if present. However, secondary Chiari conditions might not necessarily respond to a posterior cranial vault decompression [24, 25]. For that reason, a suboccipital foramen magnum decompression together with the parieto-occipital remodeling was also discussed [26–28].

In the present study, we report our extended retrospective experience with the posterior meander expansion technique regarding changes in intracranial volume, cephalic and asymmetry index, and the postoperative evolution of tonsillar position.

## Methods

### Patients

In a total of 42 children, a posterior cranial vault expansion using the posterior meander technique was performed in our center from February 2013 to October 2020. Of those, 31 children had matching sets of both preoperative and postoperative cranial MRIs available for retrospective evaluation. For all children, intracranial volume, cephalic and asymmetry index, for brachycephaly or pachycephaly and plagiocephaly, and the position of the cerebellar tonsils were calculated.

### Perioperative course

Surgical indication was based in half of the patients (21 children) on the marked head deformity, while in 16 patients it raised intracranial pressure and in 5 cases tonsillar herniation led to surgical therapy. A cranial MRI with MPRAGE sequences for neuronavigation was part of the preoperative management of all the patients. In addition, a 3D photography for digital 360° views was obtained before the operation.

**Fig. 1:**
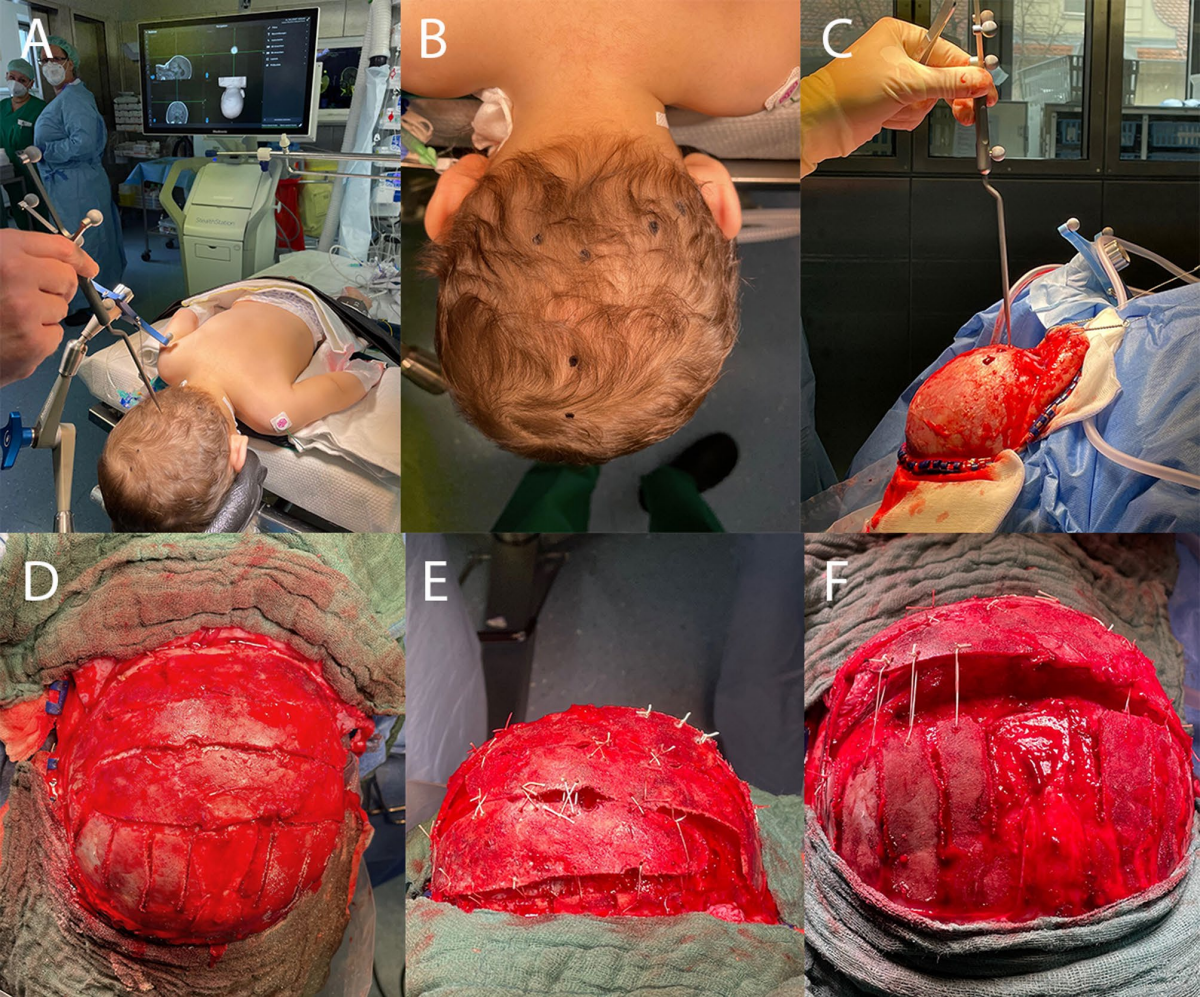
Intraoperative representative images. **A** Preoperative registration by the navigation system. **B** The sinus anatomy is depicted on the skin after registration. **C** Intraoperative identification of the sinus location on the bone by pointer navigation. **D** Meander shape bone incision and suboccipital as well as parietal barrel stave incisions. **E** View from posterior after fixation of the bone by distracting the bone fingers and applying ligation sutures at the edges accordingly. **F** View from above, indicating the volume gain by expansion

**Fig. 2:**
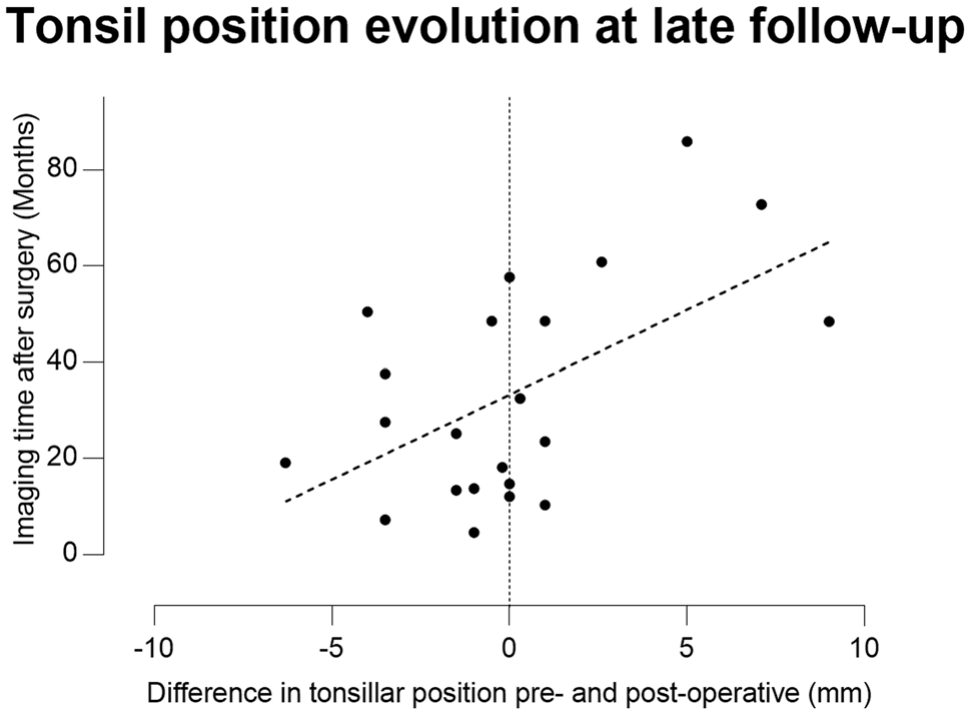
Correlation between tonsillar position change and time of imaging after surgery at late follow up (R^2^ = 0.3; p = 0.008)

Under general anesthesia, the patient was positioned prone and the head stabilized in a pediatric Mayfield or horseshoe head holder system (Doro, Integra LifeSciences, USA). Hybrid registration for neuronavigation using anatomical landmarks and surface matching was performed (Stealth 8, Medtronic, USA) in order to mark the course of the posterior superior sagittal sinus and both transverse sinus (Fig. 1). As described previously [8], a modified technique of Wagner et al. [7] was applied in all the patients: a bicoronal incision and subperiosteal and muscular dissection allowed dissection of the whole posterior skull surface including the posterior edge of the foramen magnum in the midline. After marking the course of the sagittal and transverse sinuses on the bone, parasinus burr holes and meander-shaped craniotomies were performed to create intersecting tongues with a rectangular orientation to the sagittal sinus. Extension craniotomy was done bilaterally at the tip of the tongues. Barrel stave cuts are applied to the parietal and suboccipital bone, respectively. Depending on the type of deformity, the meander shape craniotomies can be centered to the midline in brachycephalic cases or lateral in posterior plagiocephaly on the side of the lambdoid synostosis. The bony tongues were dissected from the underlying dura mater. Distraction of the tongues against each other with a significant offset and fixation of the tongues in this position using 2.0 Vicryl sutures elevated the calvarial level off the dura and increased the intracranial volume. The barrel stave craniotomies were elevated to adapt them to the level of expansion accordingly (Fig. 2). A subgaleal drainage was used and kept until the second postoperative day. Wound closure was performed with 4–0 subcutaneous Vicryl sutures and tissue glue (Dermabond, Johnson & Johnson, USA). Patients were monitored for 24–48 h postoperative in an intensive or intermediate care unit and preferably placed in a lateral position for the first couple of days. Hospital discharge was scheduled when the intravenous pain medication could be safely withdrawn.

Short-term follow-up was performed 4 weeks after surgery on outpatient appointment for clinical assessment. A cranial MRI was usually performed after 3 months postoperatively. A 3D photography was compared with the preoperative recordings.

### Measurements

The *3D intracranial volume* was calculated after segmentation of the cranium using a neuronavigation software (iPlan Cranial, version 2.6.10, Scopis, Germany or Brain Lab, Germany), based on the preoperative and the postoperative MR imaging of each patient.

The asymmetry index (AI) was calculated at four equidistant parallel levels in relation to the mid sagittal plane. Anteroposterior (AP) and biparietal (BP) diameters were obtained on the axial plane, and the lengths of the two 45° bisectors on the posterior two quadrants were used to obtain an index (the smaller bisector was the denominator). The mean among the four calculated indices was defined as the AI. The cephalic index (CI) was obtained at the level of the roof of the third ventricle.

The *tonsillar position* was detected bilaterally (left and right tonsils) in the population with symmetrical deformities, and the deepest one was used for further comparisons. The ipsilateral tonsillar position was measured in lambdoid synostosis patients. This calculation was the result of the measurement in both coronal and sagittal planes on preoperative and postoperative MRI. Negative values were reported in case of tonsillar plane above the foramen magnum. The measurements were performed from the caudal ridge of the foramen magnum.

### Statistical analysis

The analysis of the obtained data was possible through GraphPad Prism software (GraphPad 9, La Jolla, CA, USA). A statistical significance was estimated among intracranial volumes, CI and AI and tonsillar position by means of a non-parametric, paired t-test (Wilcoxon signed-rank test) with a p value < 0.05.

## Results

### Patients´ characteristics

Among the cohort of 42 children, 26 were males and 16 were females. Median age at time of surgery was 11.5 months (range 17 days to 10 years and 26 days). Nineteen presented with a symmetrical cranial deformity (brachycephaly and pachycephaly), while twenty-three were diagnosed with a posterior plagiocephaly. In particular, among the brachy- and pachycephalic children, there were 6 with Crouzon syndromes (3 reoperated), 2 pansynostotic children (one already treated elsewhere and one with a coronal, posterior sagittal, and metopic suture synostosis, later recognized as a Gorlin-Chaudhry-Moss syndrome), 2 “Mercedes-Benz” synostosis (sagittal and bilateral lambdoid suture synostosis), one craniofrontonasal syndrome, one Muenke syndrome, one osteopetrosis presenting with a sagittal suture synostosis, one bilateral coronal suture synostosis, one bilateral lambdoid suture synostosis, and one Apert syndrome. Among the anterior and/or posterior plagiocephalic children, 12 had an isolated monolateral lambdoid sutural synostosis (one later diagnosed with a Joubert syndrome), 3 were diagnosed with a pansynostosis (one “peace sign synostosis” [29] with a monolateral lambdoid suture synostosis, one child had a coronal, sagittal, and monolateral lambdoid suture synostosis, one child had a metopic, sagittal, and monolateral lambdoid suture synostosis), and 2 had combined sagittal and monolateral lambdoid suture synostosis, one Crouzon syndrome, one craniofrontonasal syndrome, one Muenke syndrome (lambdoid and coronal suture synostosis on the same side), one Pfeiffer syndrome (also a “peace sign” synostosis plus monolateral lambdoid involvement), one posterior positional plagiocephaly, and one monolateral coronal suture synostosis plus posterior plagiocephaly (Table 1).

**Table 1:** Patients characteristics (abbreviation: M—male; F—female; FOA—frontoorbital advancement; ETV – endoscopic third ventriculocisternostomy)

Number of patients		42			
Median age (months)		11.5 (range 0.6–122.5)			
Sex (M:F)		26:16			
Multisutural:monosutural		25:17			
Syndromic:non-syndromic		21:21			
Symmetric:asymmetric		23:19			
		**Total**	**Syndromic**	**Multisutural**	**Age < 1 year**
Previous surgeries (20.1 ± 30.6 months, range 2–120)	Total	30	24	24	7
	FOA	8	6	6	-
	Biparietal expansion	7	5	5	3
	Suboccipital decompression	6	5	5	1
	Shunt implantation	4	4	4	2
	Hydrocephalus surgery	4	3	3	-
	ETV	1	1	1	1
	Others	1	1	1	-
Follow-up surgeries (18.4 ± 17.3 months, range 3–72)	Total	32	28	28	22
	FOA	18	16	16	14
	Biparietal expansion	1	1	1	1
	Suboccipital decompression	6	5	5	5
	Shunt implantation	-	-	-	-
	Hydrocephalus surgery	2	2	2	-
	ETV	2	2	2	1
	Others	3	2	2	1

### Perioperative course and follow-up

Fifteen children underwent previous single or multiple surgeries: seven received one surgery, eight more than one. A fronto-orbital advancement (FOA) was performed in 8 cases, a biparietal craniectomy preceded the parieto-occipital expansion procedure in 7, and a Chiari symptomatology imposed an early suboccipital decompression before the posterior remodeling in 6. In one case, an endoscopic third-ventriculocisternostomy (ETV) was performed to establish communication between an enlarged ventricular system and the external CSF space (the child had a Crouzon syndrome and needed a ventriculo-peritoneal shunt subsequently). Four children were previously operated for a primary shunt implant. Of those, 4 shunt revisions were necessary in one child.

There were no major surgery-related complications after the posterior meander expansion cranioplasty. In one case, a localized CSF collection resolved with conservative management and in one case a confined wound dehiscence imposed a local surgical wound revision. In 39 children, an erythrocyte and/or plasma infusion (both in 25, erythrocytes in 10, and plasma in 4 children) was necessary, intra- or postoperatively (92.9%).

In 20 children, one or more surgeries followed the meander-shaped occipital expansion surgery. Ten children received only one surgery, while in the remaining 10, more than one surgery was necessary. In 18 cases, an FOA was indicated (7 received revision FOA surgeries, all syndromic cases), and in 6 a cranio-cervical decompression, in 2 an ETV (both cases did not further require a shunt), and in two a shunt revision surgery was required.

In 5 cases, the posterior expansion was done as a revision surgery. Two children were treated elsewhere previously using other techniques, and the other three children, all with Crouzon syndrome, were operated at 33.7, 35.1, and 35.4 months after the first surgery respectively (at 34.2, 45.4, and 66.8 months of age, respectively), in our Center and underwent several other surgeries, including shunt placement and revisions in between, after the first posterior advancement.

The postoperative evaluation consisted, as already mentioned, of a clinical follow-up in the outpatient clinic. The median follow-up time was 19.7 months (range 0.6–95 months). In 11 cases (26.2%), limited bone defects (maximal diameter < 2 cm) were detected at palpation at the last follow-up (all at less than 24 months after surgery). In none of the cases, an additional surgery was indicated to cover the remaining defects.

### Imaging evaluation

The mean *preoperative intracranial volume* in the general population was 1115.8 ± 327.9 cm^3^ (range 554.1–1988.6 cm^3^). The mean *postoperative intracranial* volume was significantly increased to 1356.6 ± 302.3 cm^3^ (range 783–2080.8 cm^3^) at a mean time interval of 7 ± 7.4 months after surgery (p < 0.01). The average of increase in volume from each individual postoperatively was 26.9 ± 24.1% as taken from 28 patients. The volume gain was also significant in the subgroups of children such as multisutural versus monosutural synostosis, syndromic versus non-syndromic, and children younger than 1 year versus older children (p < 0.001).

Sixteen children who received a control MRI within 6 months after surgery were calculated separately from the others with later MRIs to minimize the influence of physiologic head growth after longer MRI follow-up. In this cohort, the median age was 10 months (range 1–116 months) and the postoperative MRI was performed with a median of 4 months (range 0–6 months). In those, the mean preoperative intracranial volume was 1188.9 ± 370.4 cm^3^ and showed a significant increase in mean postoperative intracranial volume (1324.8 ± 352.9 cm^3^, p < 0.0001).

The mean *preoperative cephalic index* was 0.91 ± 0.11 which was similar to the mean *postoperative cephalic index* (0.88 ± 0.08). The mean *preoperative asymmetry index* was 0.86 ± 0.06 which showed significant increase *postoperatively* (0.91 ± 0.05, p < 0.001; Table 2).

**Table 2:** Volume changes after posterior expansion (CI: cephalic index; AI: asymmetry index; values are given as mean ± SD; *p < 0.05; **p < 0.01; ***p < 0.001 versus preoperative

		**Volume**	**CI**	**AI**
**Total**	Pre-operative	1110.8±332.7	0.91±0.11	0.86±0.06
	Post-operative	1364.5±304.8***	0.88±0.08	0.91±0.05***
	Delta	26.9±24.1%	− 0.04±0.06	0.02±0.04
**Multisuture**	Pre-operative	1056.8±351.1	0.94±0.08	0.84±0.07
	Post-operative	1332.4±307.6***	0.89±0.06**	0.89±0.06
	Delta	31.7±28.7%	− 0.05±0.07	0.02±0.04
**Monosuture**	Pre-operative	1194.2±414.0	0.73±0.12	0.88±0.05
	Post-operative	1414.1±308.1**	0.78±0.11	0.92±0.03
	Delta	21.6±12.5%	− 0.01±0.05	0.03±0.04
**Syndromic**	Pre-operative	1071.1±368.9	0.96±0.07	0.82±0.08
	Post-operative	1333.1±328.7***	0.9±0.07**	0.88±0.07
	Delta	30.2±29.4%	− 0.06±0.07	0.02±0.04
**Non-syndromic**	Pre-operative	1132.5±293.4	0.77±0.11	0.89±0.05
	Post-operative	1400.8±283.5***	0.81±0.08	0.93±0.03*
	Delta	23.2±16.5%	− 0.008±0.04	0.03±0.04
** < 1 year**	Pre-operative	964.7±283.7	0.94±0.1	0.86±0.05
	Post-operative	1295.7±300.4***	0.88±0.06**	0.92±0.03**
	Delta	38.2±27.0%	− 0.06±0.06	0.03.0±0.04
** > 1 year**	Pre-operative	1279.3±312.7	0.87±0.12	0.87±0.08
	Post-operative	1444.0±301.6***	0.87±0.1	0.9±0.07
	Delta	13.9±10.8%	− 0.02±0.05	0.01±0.04

**Fig. 3:**
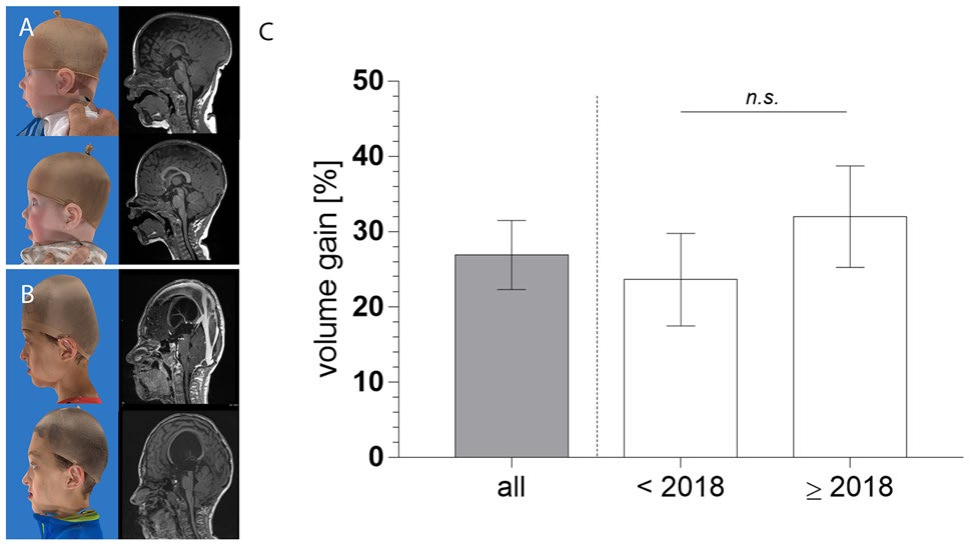
Representative examples before and after posterior meander expansion technique. **A** A 5 month-old boy with combined sagittal and bilateral lambdoid suture synostosis (“Mercedes Benz” synostosis) before and after surgery indicating lateral 3D photography and sagittal MR imaging. The patient had no previous surgery and received no further surgeries during follow up. **B** A 9.5-year-old boy, who received total cranial vault remodeling at another institution during infancy. After posterior meander expansion the posterior cranial vault is showing an improved curvature with improved tonsillar descent after surgery. No further surgery was needed in this patient. **C** Relative volume gain after posterior meander expansion achieving 26.9 ± 4.6% in the entire cohort. Before 2018, the volume gain was non-significantly lower (23.6 ± 6%) compared to the cohort operated after January 2018 (32 ± 6.7%; values are given as mean ± standard error of mean)

The tonsillar position was evaluated bilaterally in all children, and the deepest tonsillar position was used for comparison. The *mean preoperative tonsillar position* was 4.3 ± 6.8 mm below McRae’s line. Five cases showed intracranial tonsillar position, while the remaining children had a mean preoperative tonsillar position at 10.0 ± 5.8 mm. The *mean postoperative tonsillar position* in the entire cohort was 4.8 ± 6.5 mm, while in the subgroup (n = 24) with herniated tonsils it was 9.6 ± 5.4 mm. In general, there were no significant changes in mean tonsillar position between pre- and postoperative measures. However, a significant correlation between the pre- versus postoperative change in tonsillar herniation and the time to follow-up imaging could be seen (Fig. 3; Table 3).

**Table 3:** Tonsillar position measured as deepest tonsil position (values are given as mean ± standard deviation)

	Pre-operative	Post-operative	p-value (preoperative-postoperative)	Late	p-value (preoperative-late)
Total	4.3 ± 6.8	4.8 ± 6.5	p = 0.67	5.0 ± 7.1	p = 0.4
Multisutural	5.0 ± 7.0	5.5 ± 6.7	p > 0.99	5.7 ± 7.2	p = 0.87
Monosutural	0 ± 2.8	0.1 ± 3.0	p = 0.5	− 0.6 ± 3.4	p = 0.12
Syndromic	5.4 ± 7.5	5.9 ± 7.1	p = 0.67	6.2 ± 7.6	p = 0.67
Non-syndromic	1.1 ± 2.5	1.6 ± 2.9	p = 0.46	1.1 ± 2.9	p = 0.12
< 1 year	1.0 ± 4.8	2.6 ± 6.2	p = 0.12	2.9 ± 6.5	p = 0.75
> 1 year	8.1 ± 7.3	7.6 ± 6.3	p = 0.18	7.5 ± 7.4	p = 0.20

## Discussion

Our series portrays the course of 42 children with brachy-/pachy- or plagiocephaly belonging to a syndromic (50%) as well as to a non-syndromic group (50%). In particular for the syndromic population, an early posterior expansion may reduce the need for a further fronto-orbital advancement [13]. In our series, the 66.7% (14 out of 21 syndromic cases) of our syndromic children required a surgical intervention to further expand the intracranial volume. For 6 children (28.9%), the frontal remodeling was performed before the parieto-occipital expansion. One child did not require any further surgeries.

The *surgical indication* differs among the two populations: the main issue in the syndromic group is to reach an acceptable gain in intracranial volume, to resolve or improve a tonsillar descent and to decrease the possibly elevated intracranial pressure. In the non-syndromic population, the main surgical goal is usually to reach a good cosmetic result, towards a more symmetrical posterior cranial shape, thereby increasing the unilateral posterior fossa occipital volume.

Distractive osteogenetic *techniques* are usually compared to osteoplastic techniques, which include the presented meander-technique cranioplasty. Most other described osteoplastic techniques implement a complete mobilization of a posterior, parieto-occipital bone flap. In contrast, the meander cranioplasty utilizes the parieto-occipital calvarium to create intersecting calvarial tongues to distract and stabilize without heterologous materials. The avoidance of surgeries to explant distraction devices, the possible early mobilization of the child, and the reduced risk of major bone lacunae are the most important advantages of the presented technique.

In terms of *complications*, according to our experience, 4.8% of the children (2 out of 42) were affected by either a CSF collection, which resolved without the need of surgery, or a wound healing disturbance, which had to be surgically treated. Other remodeling techniques such as those reported by Eibach et al. using the “Pantheon” technique observed no relevant complications in a series of 121 procedures [5]. On other smaller series, some sporadic reoperations were performed because of correction loss in 3 out of 25 children [3, 27]. These rates appears to be decent in comparison to distractive methods, as demonstrated by a review by Greives et al. (22.6%) [30]. The technique of osteogenic distraction appears to be associated with higher rates of infection (7.4–29%) [1, 17, 22, 25, 28, 31, 32], failure of the implants used for the fixation with loosening or dislocation of its components (0–12%) [1, 32, 33], and dural tears (6.5–10%) [30–32]. A possible advantage of the distraction technique is the shorter surgery time and a limited blood loss with consecutive lower transfusion rate [17]. A few comparative series between distractive and remodeling techniques have been published, also indicating infection as a more frequent postoperative issue in distraction procedures (7.4%), while in the remodeling technique, fractures and hematomas have been more often encountered [17, 22]. The rate of reoperations was 7.1% after the posterior meander expansion technique. Two out of three children were younger than 1 year at the time of the first surgery, and all of them were affected by Crouzon syndrome and were operated in the first half of children before 2018. This rate is comparable to the series of Fearon et al., with 3 cases of reoperation in a series of 25 children [27].

Concerning the *gain in intracranial volume*, a few results after posterior cranial vault distraction were published in the last few years. Some authors reported their outcomes in terms of volume only on distracted children, in a range of 10.2% and 28.5% volume gain [18, 19, 25]. In studies in which both techniques were compared, 25% volume increase in the distraction technique could be achieved, while posterior remodeling showed up to 29% [11, 17]. Those volumetric results reveal similar results compared to our cohort being 26.9%. Interestingly, the volume gain was higher in the second part of our cohort after January 2018 (32%) compared to the earlier half before 2018 (23.6%), indicating some learning curve with the posterior meander expansion technique. Further comparative studies between the two techniques are needed in order to assess the real advantages and disadvantages of different methods.

Interestingly, in our population, the cephalic index was the only parameter which did not show significant differences after surgery in the entire cohort. In a more detailed analysis, syndromic and multisutural infants showed significant reduction of cephalic index after surgery towards a more normal value. In the remaining cohort, the CI values remain unchanged. Thus, the more severe deformities showed a more pronounced brachycephaly and did profit more significantly towards a better proportion of the cephalic index. Tonsillar herniation was observed not in all of the children and, if present, did improve inconstantly after the surgery. The tonsillar herniation was more pronounced in syndromic, multisutural, and older children, while a tendency towards improvement could only be seen in older children. Two factors may be responsible for this finding. First is the ongoing dynamic changes which are present even after surgery especially if the children have complex malformations or are still very young, and secondly, the expansion technique of the occipito-parietal vault does not affect too much the tonsillar herniation by itself. This has also been reported previously by others using the same [27] and other techniques [25, 34]. Thus, it was suggested to perform the expansion together with a decompression of the craniocervical junction in the same session [24]. Di Rocco et al. described a good resolution of *Chiari symptomatology*, but not necessarily an early radiological improvement, similarly to our results [25]. Interestingly, later MRI follow-ups showed actually some radiological improvement of the tonsillar descent (Fig. 3). In our experience, 11.9% already received an osteodural decompression through a suboccipital approach before, while 14.3% were operated later (3 belonged to the early operated group). Our more recent strategy is to decompress the medial portion of the suboccipital barrel stave in order to achieve a craniocervical decompression within one surgery during posterior meander expansion technique.

## Conclusion

Our extended experience over a decent period of the past years has enabled us to state that parieto-occipital expansion by means of posterior meander technique is effective in terms of volume gain and safe in terms of low complication rate and postoperative calvarial stability and reossification and is very well comparable with other techniques. The main advantage is represented in the fact that no implants are necessary to achieve decent results. Limitations are mostly related to the ongoing dynamic changes during impaired growth especially in the severely affected children. Further collaborative studies are needed for these rare conditions in order to further improve the surgical strategies.
